# Effectiveness of powered exoskeleton use on gait in individuals with cerebral palsy: A systematic review

**DOI:** 10.1371/journal.pone.0252193

**Published:** 2021-05-26

**Authors:** Lucinda Rose Bunge, Ashleigh Jade Davidson, Benita Roslyn Helmore, Aleksandra Daniella Mavrandonis, Thomas David Page, Tegan Rochelle Schuster-Bayly, Saravana Kumar

**Affiliations:** UniSA Allied Health & Human Performance, University of South Australia, Adelaide, Australia; BG-Universitatsklinikum Bergmannsheil, Ruhr-Universitat Bochum, GERMANY

## Abstract

**Background:**

Cerebral palsy (CP) is a leading cause of childhood disability. The motor impairments of individuals with CP significantly affect the kinematics of an efficient gait pattern. Robotic therapies have become increasingly popular as an intervention to address this. Powered lower limb exoskeletons (PoLLE) are a novel form of robotic therapy that allow the individual to perform over-ground gait training and yet its effectiveness for CP is unknown.

**Purpose:**

To determine the effectiveness of PoLLE use on gait in individuals with CP.

**Method:**

A systematic search of eight electronic databases was conducted in March 2020. Studies included children (0–18 years) and or adults (18+ years) diagnosed with CP who used a PoLLE for gait training. This review was conducted and reported in line with the Preferred Reporting Items for Systematic Review and Meta-Analyses (PRISMA) statement, with the methodology registered with PROSPERO (CRD42020177160). A modified version of the McMaster critical review form for quantitative studies was used to assess the methodological quality. Due to the heterogeneity of the included studies, a descriptive synthesis using the National Health & Medical Research Council (NHMRC) FORM framework was undertaken.

**Results:**

Of the 2089 studies screened, ten case series and three case studies met the inclusion criteria highlighting the current evidence base is emerging and low level. A range of PoLLEs were investigated with effectiveness measured by using a number of outcome measures. Collectively, the body of evidence indicates there is some consistent positive evidence on the effectiveness of PoLLE in improving gait in individuals with CP, with minimal adverse effects. While this is a positive and encouraging finding for an emerging technology, methodological concerns also need to be acknowledged.

**Conclusion:**

With rapidly evolving technology, PoLLEs could play a transformative role in the lives of people impacted by CP. Ongoing research is required to further strengthen the evidence base and address current methodological concerns.

## Background

Cerebral palsy (CP) refers to a group of permanent, non-progressive neurological disorders occurring in the development of the foetal or infant brain, primarily affecting movement and posture [1, 2]. It is estimated that 17 million individuals have CP globally [3]. The incidence rate of CP is high in many nations. The incidence rates per 1000 live births were 1.4 in Australia during 2010–2012 [4], 2.08 in Europe during 1980–1990 [5], 3.2 in the United States during 2009–2016 [6], and varying statistics amongst developing countries with Uganda recording 2.7–3.1 during 2015 [7].The life expectancy of individuals with this disorder varies according to the level of disability. An individual with mild CP may have a life expectancy equal to or slightly lower than that of the general population [8]. However, individuals with severe CP may have a significantly shortened lifespan of approximately 20% less than the general population [8].

The level of CP is categorised into five different grades from I to V based on the Gross Motor Function Classification System (GMFCS) [9]. All individuals with CP have motor impairments, while only some may have intellectual, speech, hearing, and visual impairments, as well as other comorbidities such as epilepsy [10]. The motor impairments often seen in individuals with CP include muscle weakness, abnormal muscle tone, contractures, and fatigue, all of which can significantly affect the kinematics of an efficient gait pattern (e.g. step length, gait velocity etc.) [1]. As such, therapeutic interventions are often targeted at improving motor impairments to maximise gait efficiency and independence [11].

Management focuses on addressing motor impairments to optimise an individual’s function and quality of life as there is no cure for CP [2]. Typical interventions to address motor impairments include botulinum toxin injections, serial casting, orthopaedic surgery, task-specific training, orthoses, strength training, stretching, hydrotherapy and home exercise programs [12, 13]. Therapy should also incorporate cognitive engagement and massed practice [14]. The mental connection to the motor learning, or cognitive engagement, enhances the potential of neuroplasticity [15]. Massed practice describes a training style consisting of fewer and shorter breaks during a single training session to optimise motor learning [16]. It can be difficult to achieve the massed practice required in a therapy session due to the physical demands placed on therapists [14]. Increasingly, robotic therapies are being considered in the management of individuals with CP as one way to address this challenge. Robotic-assisted gait training refers to devices that use robotic exoskeletons or footplates to assist in the guidance of lower limb movements in the gait cycle [11]. Robotic-assisted gait training can provide cognitively engaging massed practice of gait that is less physically demanding on therapists [14].

To date, much of the literature has focused on driven gait orthoses, including the Lokomat, ReoAmbulator and Gait Trainer GT 1 [11, 14]. A systematic review by Lefmann, Russo & Hillier [11] and Carvalho et al. [1] reported that driven gait orthoses may have a positive effect on gait in people with grade I-IV CP, while acknowledging the low level evidence amongst the included studies. In contrast, passive and powered lower limb exoskeletons are a novel form of robotic-assisted gait training that have commercially emerged in the last ten years [17], enabling over-ground walking, independent of a treadmill system [13]. Powered lower limb exoskeletons (PoLLE) consist of an external, powered, motorised orthosis that is placed over a person’s paralysed or weakened limbs for medical purposes [18], and is produced under brand names such as ReWalk, HAL, Ekso and Indego [17]. In contrast, passive lower limb exoskeletons are not motorised, operating through reducing the force required to generate movement at the lower limbs [19]. The motorised component of a PoLLE enables individuals to execute a more normal gait pattern, in comparison to a compensatory and inefficient gait pattern with passive lower limb exoskeletons, as found in stroke patients [19].

While there is a growing body of evidence on the effectiveness of PoLLE in spinal cord injury [20–23], stroke [24, 25] and neurological disorders broadly [17], the evidence for CP is unknown. Therefore, the aim of this systematic review was to summarise the current literature on the evidence of the effectiveness of PoLLE use on gait in individuals with CP for spatiotemporal parameters, energy expenditure and safety.

## Methods

The protocol for this review was registered with The International Prospective Register of Systematic Reviews (PROSPERO) (registration number: CRD42020177160). This review followed the Preferred Reporting Items for Systematic Reviews and Meta-Analyses (PRISMA) statement [26] and checklist (S1 Appendix).

### Search strategy

The PICO research format was utilised in the development of the search strategy, which included keywords in relation to the population and intervention of interest. Discussions with The University of South Australia Academic Librarian helped inform the final search strategy to increase sensitivity and comprehensiveness. On the 24th of March 2020, all eight electronic databases were searched with no date restrictions. The primary databases included Medline, Embase, Emcare, and Scopus. Secondary databases were The Cochrane Library, IEEE Xplore, Physiotherapy Evidence Database and OTSeeker. The databases selected provide international, peer-reviewed research on biomedical and multi-disciplinary healthcare. The IEEE Xplore database also has a specific focus on engineering and technology literature and was chosen to capture a comprehensive search in robotics and exoskeletons. The search strategy was initially developed using Medline, which included a combination of key words and MeSH terms. The complete search strategy for Medline is detailed in S2 Appendix with the search syntax included in S3 Appendix. Slight modifications were then made to the search strategy to accommodate individual database requirements (for example, using Emtree instead of MeSH for Emcare). Once relevant literature was identified, their reference lists were searched to identify additional studies (Pearling). Grey literature was then searched through Google, with the first 100 results reviewed [27]. Websites of relevant PoLLE manufacturers (Ekso, ReWalk, Indego and HAL) were also searched for further research studies.

### Study design

The review included all primary quantitative studies. According to the National Health and Medical Research Council (NHMRC) [28], this includes all study designs listed from Levels II-IV under the intervention category.

### Population

Studies were included if participants were children (0–18 years) and or adults (18+ years) who have a diagnosis of CP, either unilateral or bilateral, by a healthcare professional. For this review, any functional level of CP from I-V based on the GMFCS was included. Studies were excluded if participant’s gait impairments were not caused by their CP diagnosis.

### Intervention

Studies were included when the intervention for review was a PoLLE, used either as a stand-alone treatment, or as an adjunct to standard healthcare. A PoLLE was defined as an over-ground wearable robotic unit that can also be identified by brand name, such as Ekso and REX. Standard healthcare for CP can be delivered by numerous health professionals such as physiotherapists, occupational therapists and general practitioners, and includes but is not limited to strength training, orthotics, gait training and hydrotherapy. The PoLLE must have been used for bipedal gait training in participants experiencing motor impairments associated with their walking due to their CP diagnosis. Studies were excluded when robotic-assisted gait training was used on a treadmill or in conjunction with exergaming or virtual reality training.

### Comparator

The comparator for included studies was any kind of control intervention including standard healthcare for gait disorders in individuals with CP and no intervention. Standard care represents treatment as usual, and includes any treatment offered for gait disorders. This excludes interventions for upper limb function, communication, eating and drinking and bladder and bowel control.

### Outcome measures

The primary outcome of interest was gait parameters, which can be measured through step length, stride length, cadence and mean velocity. Secondary outcomes included energy expenditure and safety. Energy expenditure was measured using the Physiological Cost Index (PCI), energy cost, metabolic cost and the Borg scale. Measures of safety included any reports of adverse events, such as skin irritation, abrasions and falls.

### Literature search

Two reviewers (AD, TP) independently ran each database search to ensure reproducibility and reliability. Results from each database search were exported into Endnote^TM^, a bibliographic software tool used to store and manage citations. This library was then uploaded to Covidence^TM^, a cloud-based platform that enabled the removal of duplicates and independent screening by multiple reviewers. Two reviewers (AM, BH) independently screened the preliminary list of studies by title and abstract in conjunction with the inclusion and exclusion criteria. The subsequent full text studies were then independently analysed to determine their eligibility based on the PICO criteria. Discrepancies were resolved by discussion with a third reviewer (SK), where required.

### Methodological quality assessment

The included studies were ranked independently by all six reviewers using the intervention category from the NHMRC Evidence Hierarchy [28]. Disagreements were resolved by discussion, or through consultation with another reviewer (SK) where required. To assess methodological quality, a modified version of the quantitative McMasters Critical Appraisal Tool (MMCAT) [29] was used. This critical appraisal tool was chosen as it is a published, freely available, widely used tool in systematic reviews [21, 30] and can be used across a range of quantitiative study designs. The MMCAT assessed a range of domains as part of the appraisal process including purpose, literature review, design, sampling, outcomes, intervention, results and conclusion with implications for practice. While the original MMCAT does not provide a numerical score as part of the critical appraisal process, in order to compare the metholodological quality within and across the included studies, the original tool was modified to include scoring. Each criterion was rated as ‘yes’, ‘no’, ‘not addressed’ or ‘N/A–not applicable’. One point was awarded for every ‘yes’ answer, while no points were awarding for ‘no’ or ‘not addressed’. Overall, the minimum score was zero and the maximum score was 14. In some instances, the ‘N/A’ criterion was selected (as the criterion did not apply to some study designs), which then altered the overall scoring. Therefore, the overall scores for individual studies were converted to percentages for ease of interpretation and comparison purposes. Four studies [31–34] were independently rated by all reviewers to establish reliability and consistency of the appriasal process. Following this, each study was independently rated by two independent reviewers from the review team, with disagreements resolved by discussion or in consultation with a third reviewer where required.

### Data extraction

A customised data extraction form was developed by the reviewers (TP, TSB) using Microsoft Word^TM^. The data extraction form contained key elements on study design, population characteristics, sample size, intervention type, duration and frequency, outcome measures and results on spatiotemporal parameters, energy expenditure and safety (in particular, statistical analysis including mean and standard deviation). The extracted data items were informed by the PICO framework and chosen as means to adequately answer the review question. The data extraction form was reviewed by an experienced reviewer (SK) to ensure all relevant data were captured. All six reviewers independently extracted data from two studies [31, 32] to ensure reliability and consistency, after which two independent reviewers (TP, TSB) extracted the data from the remaining studies. Discrepancies were resolved through discussion and consultation with the third reviewer (SK) where needed.

### Data synthesis

Due to the heterogeneity amongst the included studies, a meta-analysis was not performed. Rather, a narrative synthesis of all relevant literature was conducted using the NHMRC FORM [35] framework to assist the process of synthesising the evidence from the available literature. This framework has been successfully used in previous systematic reviews [36, 37]. The NHMRC FORM framework is underpinned by five key components, each rated from excellent to poor, namely (A) evidence base, (B) consistency, (C) clinical impact, (D) generalisability and (e) applicability to the Australian health care setting [35]. Applicability was not used in this review as findings may be applicable to a broader and diverse international context. All studies were synthesised independently by the six reviewers who provided a rating for sections (A) to (D). The ratings for each section were compared between the reviewers to ensure consistency and repeatability. An experienced reviewer (SK) then verified these ratings subsequent to which all reviewers, as a collective, developed a final grade of recommendation.

## Results

### Search results

The search strategy generated 2935 results, with an additional 17 records identified through other grey literature sources (such as manufacturers’ websites and Google). Following the removal of duplicates, 2089 studies were screened based on title and abstract. The full text of 109 studies were reviewed, and 17 studies successfully met the eligibility criteria and were included in this review. Even though the screening process identified 17 studies, the final number of included studies was 13. There were two reasons for this. First, as two studies [38, 39] reported data on the same participant, the data from these two studies were amalgamated and considered as a single study. Second, in three separate instances, two studies shared the same participants [31, 38–42]. In these cases, the study with the larger sample size, and hence more comprehensive data, was included in this review.

**Fig 1:**
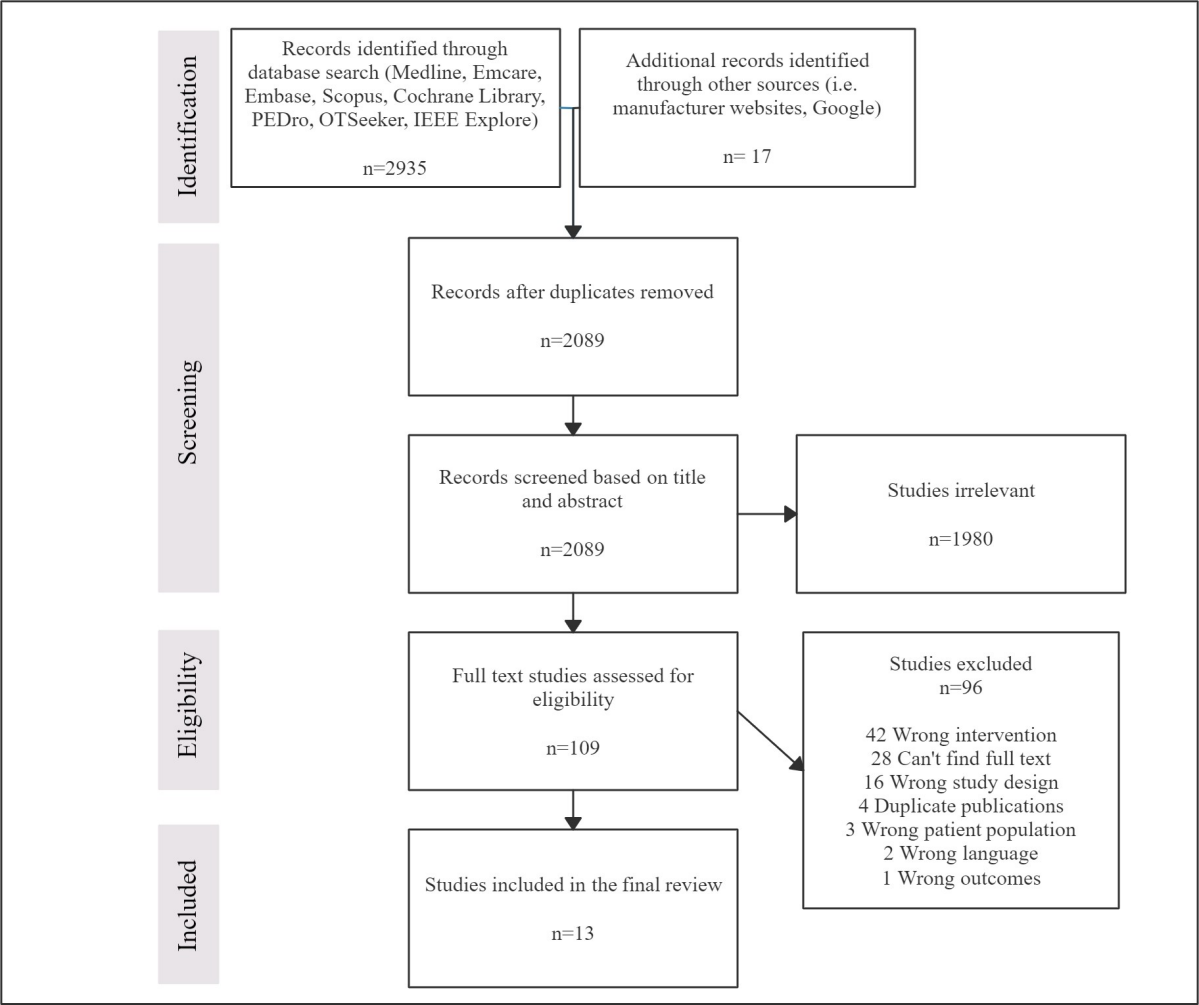
PRISMA flowchart.

Other reasons for exclusion included wrong intervention, no full text available and wrong study design. No further studies were identified from pearling the reference lists. Fig 1 provides an overview of the literature selection process.

### Methodological quality

Table 1 provides an overview of the methodological quality and the level of evidence of the included studies. Using the NHMRC levels of evidence [28] all of the included studies were ranked as Level IV evidence. Ten studies were classified as ‘case series’ [31–33, 41–47] and three studies as ‘case studies’ [34, 39, 48].

**Table 1:** Levels of evidence and critical appraisal scores.

Study	NHMRC level and study design	Items on modified McMaster critical appraisal tool														Raw score and %
		1	2	3	4	5	6	7	8	9	10	11	12	13	14	
Bayon et al. (2016) [42]	IV; Case series	1	1	1	N/A	0	0	1	N/A	0	0	0	0	1	1	6/12 50.0%
Bayon et al. (2018) [31]	IV; Case series	1	1	1	N/A	0	0	1	N/A	0	0	0	1	1	1	7/12 58.3%
Lerner, Damiano, Bulea (2017) [32]	IV; Case series	1	1	1	N/A	0	0	0	N/A	0	1	1	0	1	1	7/12 58.3%
Mataki et al. (2018) [48]	IV; Case study	0	1	1	N/A	0	0	1	N/A	0	0	0	0	N/A	1	4/11 36.4%
Matsuda et al. (2018a) [47]	IV; Case series	1	1	1	N/A	0	0	1	N/A	N/A	1	1	0	0	0	6/11 54.5%
Matsuda et al. (2018b) [46]	IV; Case series	1	1	1	N/A	0	0	1	N/A	0	1	1	0	1	1	8/12 66.7%
Mileti et al. (2016) [45]	IV; Case series	1	1	1	N/A	0	0	1	N/A	N/A	1	1	0	1	1	8/11 72.7%
Nakagawa et al. (2019a) [39]	IV; Case study	1	1	1	N/A	0	0	1	N/A	0	0	0	0	N/A	1	5/11 45.5%
Nakagawa et al. (2019b) [41]	IV; Case series	1	1	1	N/A	0	0	1	N/A	N/A	1	1	0	1	1	8/11 72.7%
Orekhov et al. (2020) [33]	IV; Case series	1	1	1	N/A	0	0	1	N/A	0	1	1	0	0	1	7/12 58.3%
Smania et al. (2011) [34]	IV; Case study	1	1	1	N/A	1	1	1	N/A	N/A	0	1	0	N/A	1	8/10 80.0%
Takahashi et al. (2018) [44]	IV; Case series	1	1	1	N/A	0	0	0	N/A	N/A	1	1	0	1	1	7/11 63.6%
Ueno et al. (2019) [43]	IV; Case series	1	1	1	N/A	0	0	1	N/A	0	1	1	0	1	1	8/12 66.7%

MMCAT items to be scored: 1. Was the purpose stated clearly?; 2. Was relevant background literature reviewed?; 3. Was the sample described in detail?; 4. Was the sample size justified?; 5. Were the outcome measures reliable?; 6. Were the outcome measures valid?; 7. Intervention was described in detail?; 8. Contamination was avoided?; 9. Cointervention was avoided?; 10. Results were reported in terms of statistical significance?; 11. Were the analysis method/s appropriate?; 12. Clinical importance was reported?; 13. Drop-outs were reported?; 14. Conclusions were appropriate given study methods and results?. 1 = yes, 0 = no or not addressed, N/A = not applicable.

The methodological quality of the included studies was considered as poor to moderate. The raw scores were converted to a percentage for ease of comparison across the included studies as some criteria did not apply to all study designs. For example, the criterion for co-intervention was not applicable to the studies which only had a single intervention session of robotic therapy [34, 41, 44–46]. Similarly, given the included studies were either case series or case studies, there were no control or comparator groups which meant the criterion for contamination was not applicable across all studies. The highest critical appraisal score was awarded to Smania et al. 34] and the lowest to Mataki et al. [48]. While most of the included studies scored well for criterion two [31–34, 39, 41–48] and criterion 14 [31–34, 39, 41–45, 47, 48], there were a number of methodological concerns. These included measurement bias due to lack of psychometrically sound outcome measures [31–33, 39, 41–48], co-intervention bias [31–33, 39, 42, 43, 47, 48] and lack of appropriate analytical methods [31, 39, 42, 48]. While the lack of blinding of the participants, therapists and measures increases the risk of placebo, Hawthorne effect and measurement bias, given the nature of the intervention, these biases could not be entirely avoidable.

### Study characteristics

Table 2 highlights characteristics of all included studies. Studies were published between 2011–2020, with a large concentration of studies originating in Japan (n = 7) with Spain, Italy and the United States contributing two studies each.

**Table 2:** Study characteristics.

Study	Design	n	Population Characteristics	Intervention	Comparator	Outcomes	Results	Main Findings
**Bayon et al. (2016) Spain [42]**	Case Series	2	Ages: 12 and 14yrs M: Not reported F: Not reported Diagnosis: Spastic Diplegia CP GMFCS: Level II: 1 Level III: 1 Co-Intervention: No	Exoskeleton: CP Walker robotic platform n Rx = 10 5/52	N/A	**Spatiotemporal** • 22Cadence • Gait Velocity • Step length	**Spatiotemporal** (pre-post) Gait Velocity (m/s) P1: Pre: 0.40±0 Post: 0.49±0 P2: Pre: 0.60 ±.10 Post: 0.80±0 Cadence (step/min) P1: Pre: 73.80 ± 6.00 Post: 75.80 ± 7.97 P2: Pre: 102.20 ± 12.65 Post: 120.8 ± 9.38 Step Length (m) P1: Pre: L) 0.24 ± .04 Pre: R) 0.30 ± .01 Post: L) 0.27 ± .01 Post: R) 0.33 ± .02 P2: Pre: L) 0.24 ± .05 Pre: R) 0.31 ±.03 Post: L) 0.38 ± .02 Post: R) 0.40 ± .01 No reporting of P Values	CPWalker use in robot-based training program may improve gait in children with CP
**Bayon et al. (2018)** **Spain [31]**	Case Series	4	Age Range: 12-17yrs F: 2 M: 2 Diagnosis: Spastic CP GMFCS: Level II: 2 Level III: 2 Co-Intervention: No	Exoskeleton: CPWalker robotic platform n Rx = 16 8/52	N/A	**Spatiotemporal** • Mean Velocity • Cadence • Step Length **PCI**	**Spatiotemporal** • Collective mean velocity: 21.46% +/- 33.79% average increase • Collective cadence: 2.84% +/- 13.96% average increase • Collective step length: 17.95% +/- 20.45% average increase **PCI** • P1: 0.75 beats/m and 0.55 beats/m for middle and post assessments respectively • P2: 0.89 beats/m and 0.80 beats/m for middle and post assessments respectively • P3: 1.57 beats/m and 1.26 beats/m for middle and post assessments respectively • P4: 0.33 beats/m and 0.03 beats/m for middle and post assessments respectively No reporting of P Values	CPWalker use in robot-based training program may improve gait in children and adolescents with CP
**Lerner, Damiano, Bulea (2017) USA [32]**	Case Series	7	Age Range: 5-19yrs F: 3 M: 4 Diagnosis: CP with crouch gait GMFCS: Level I: 1 Level II: 6 Co-Intervention: No	Exoskeleton: Wearable Exoskeleton n Rx = 6 8-12/52	N/A	**Spatiotemporal** • Step length • Cadence • Gait velocity	Walking with powered exoskeleton versus baseline at final assessment **Spatiotemporal** • Step length: No significant change (more-affected limb, P = 0.22; less-affected limb, P = 0.22) • Cadence: No significant change (more-affected limb, P = 0.07; less-affected limb, P = 0.17) • Gait velocity: No significant change (P = 0.05)	Powered exoskeleton did not significantly reduce crouch gait in children and adolescents with CP
**Mataki et al. (2018)** **Japan [48]**	Case Study	1	Age: 15 yrs F: 0 M: 1 Diagnosis: Spastic Diplegia CP GMFCS: Level IV: 1 Co-Intervention: post-surgery for tendon lengthening of bilateral hamstrings and Achilles tendons	Exoskeleton: The Hybrid Assistive Limb (HAL) CYBERDYNE n Rx = 2 during postoperative months 10 and 11	N/A	**Spatiotemporal** • Stride length • Cadence • Gait speed	(pre-post)**Spatiotemporal** • Stride: Increased from 0.40 to 0.41m/step after 1^st^ HAL intervention • Stride: Increased from 0.45 to 0.47m/step after 2^nd^ HAL intervention • Cadence: Increased from 54.28 to 58.8 steps/min after 1^st^ HAL intervention • Cadence: Increased from 59.81 to 68.86 steps/min after 2^nd^ HAL intervention • Gait speed: Increased from 21.71 to 24.00m/min after 1^st^ HAL intervention • Gait speed: Increased from 26.71 to 36.05m/min after 2^nd^ HAL intervention No reporting of P Values	HAL use is likely to be safe and may improve gait in individuals with CP
**Matsuda et al. (2018a) Japan [46]**	Case Series	12	Mean Age: 16.2 yrs F: 4 M: 8 Diagnosis: Spastic Diplegia: 9 Spastic Quadriplegia: 1 Spastic Right Hemiplegia: 2 GMFCS: Level I: 2 Level II: 3 Level III: 5 Level IV: 2 Co-Intervention: No	Exoskeleton: The Hybrid Assistive Limb (HAL) CYBERDYNE n Rx = 1 mins/day = 10–20	N/A	**Spatiotemporal** • Stride length • Cadence • Gait speed	**Spatiotemporal** (pre-post) *p<0.05, **p<0.01. Gait speed (m/s) Pre: 0.8 ± 0.4 During: 0.3 ± 0.2** Post: 0.8 ± 0.5 Stride length (m) Pre: 0.5 ± 0.1 During: 0.4 ± 0.1* Post: 0.5 ± 0.1 Cadence (step/min) Pre: 92.4 ± 35.9 During: 55.1 ± 17.5** Post: 92.4 ± 40.0	Single HAL use did not improve spatiotemporal gait parameters in individuals with CP
**Matsuda et al. (2018b) Japan [47]**	Case Series	6	Mean Age: 16.8 yrs F: 2 M: 4 Diagnosis: Spastic Diplegia (5) and Spastic Quadriplegia (1) CP types GMFCS: Level II: 1 Level III: 4 Level IV: 1 Co-intervention: Usual Physiotherapy	Exoskeleton: The Hybrid Assistive Limb (HAL) CYBERDYNE n Rx = 12 4/52	N/A	**Spatiotemporal** • Step length • Cadence • Gait speed **PCI**	**Spatiotemporal: SWS** (n = 6) (pre-post) Gait speed (m/s) Pre: 0.5 ± 0.3 Post: 0.7 ± 0.5 P = 0.046* Mean step length (cm) Pre: 39.4 ± 10.2 Post: 45.0 ± 13.1 P = 0.078 Cadence (min/step) Pre: 72.7 ± 30.2 Post: 89.9 ± 39.4 P = 0.013* Spatiotemporal: MWS (n = 5) (pre-post) Gait speed (m/s) Pre: 0.8 ± 0.3 Post: 1.0 ± 0.5 P = 0.104 Mean step length (cm) Pre: 48.7 ± 9.5 Post: 50.0 ± 9.9 P = 0.739 Cadence (min/step) Pre: 93.3 ± 40.9 Post: 110.4 ± 50.1 P = 0.028* PCI (beat/m) (n = 5) (pre-post) Pre: 1.8 ± 0.7 Post: 1.6 ± 0.3 P = 0.405	HAL use is likely to be safe and may improve gait in adolescents and young adults with CP
**Mileti et al. (2016) Italy [45]**	Case Series	3	Mean Age: 11.0 yrs F: 2 M: 1 Diagnosis: Right hemiplegia CP GMFCS: Not specified Co-Intervention: No	Exoskeleton: WAKE-up (Wearable Ankle Knee Exoskeleton) n Rx = 1 mins/day = 30	Compared to: n: 4 Mean Age: 10.5 yrs Diagnosis: N/A (Healthy)	**Spatiotemporal** • Cadence • Gait speed • Stride length • Step length	**Spatiotemporal** Cadence (Cad [step/min]) P1 Pre: L: 87.1 (5.3) Post: L: 73.0 (6.7) P = 0.05* Pre: R: 86.4 (3.3) Post: R: 71.8 (2.5) P = <0.01** P2 Pre: L: 84.4 (3.4) Post: L: 72.7 (1.9) P = <0.01** Pre: R: 84.1 (1.1) Post: R: 75.3 (3.2) P = <0.01** P3 Pre: L: 110.2 (7.6) Post: L: 106.0 (10.5) P = 0.04* Pre: R: 115.9 (15.5) Post: R: 107.7 (13.9) P = 0.21 **Walking Speed** (WS [m/s]) **P1** Pre: L: 0.7 (0.0) Post: L: 0.6 (0.1) P = 0.01** Pre: R: 0.7 (0.0) Post: R: 0.6 (0.1) P = <0.01** P2 Pre: L: 0.4 (0.0) Post: L: 0.4 (0.0) P = 0.01** Pre: R: 0.3 (0.0) Post: R: 0.4 (0.0) P = <0.01** P3 Pre: L: 0.5 (0.1) Post: L: 0.6 (0.1) P = 0.15 Pre: R: 0.5 (0.1) Post: R: 0.5 (0.1) P = 0.21 **Stride Length** (SL [m]) **P1** Pre: L: 1.0 (0.1) Post: L: 1.0 (0.1) P = 0.77 Pre: R: 1.0 (0.1) Post: R: 1.0 (0.1) P = 0.33 **P2** Pre: L: 0.5 (0.1) Post: L: 0.7 (0.3) P = <0.01** Pre: R: 0.5 (0.0) Post: R: 0.6 (0.1) P = <0.01** P3 Pre: L: 0.5 (0.1) Post: L: 0.6 (0.1) P = 0.01** Pre: R: 0.5 (0.1) Post: R: 0.6 (0.1) P = 0.03* Step Length (SpL [m]) **P1** Pre: L: 0.5 (0.1) Post: L: 0.5 (0.1) P = 0.83 Pre: R: 0.5 (0.1) Post: R: 0.5 (0.1) P = 0.48 **P2** Pre: L: 0.2 (0.1) Post: L: 0.3 (0.0) P = 0.01** Pre: R: 0.3 (0.0) Post: R: 0.4 (0.0) P = <0.01** P3 Pre: L: 0.3 (0.1) Post: L: 0.4 (0.0) P = 0.03* Pre: R: 0.2 (0.0) Post: R: 0.3 (0.0) P = 0.01**	Wearing WAKE-up exoskeleton may improve gait in children with CP
**Nakagawa et al. (2019a) Japan [39]**	Case Study	1	Age: 17 yrs Male F: 0 M: 1 Diagnosis: Spastic Diplegia GMFCS: Level IV: 1 Co-Intervention: Daily Physiotherapy	Exoskeleton: The Hybrid Assistive Limb (HAL) CYBERDYNE n Rx = 12 4/52	N/A	**Spatiotemporal** • Step length • Gait speed **Borg Scale**	**Spatiotemporal** Step length (m) • Pre-training: R) 0.29 & L) 0.30 • Immediate post-training: R) 0.26 & L) 0.21 • 1-month post-training: R) 0.32 & L) 0.41 • 4 months post-training: R) 0.39 & L) 0.33 • 7 months post-training: R) 0.39 & L) 0.31 Gait speed (m/min) • Pre-training: 17.9 • Immediate post-training: 14.2 • 1-month post-training: 21.9 • 4 months post-training: 21.5 • 7 months post-training: 21.2 **Borg Scale** • Mean: 11.9 • Range: 11–13 No reporting of P Values	HAL use is likely to be safe and may produce sustained gait improvement in post-pubertal individuals with CP
**Nakagawa et al. (2019b) Japan [41]**	Case Series	19	Mean Age: 8.5 F: 6 M: 13 Diagnosis: Spastic Diplegia: 15 Spastic Hemiplegia:1 Ataxia: 2 Athetosis: 1 GMFCS: Level I: 2 Level II: 2 Level III: 8 Level IV: 7 Co-Intervention: Usual Physiotherapy	Exoskeleton: The Hybrid Assistive Limb (HAL) CYBERDYNE n Rx = 1 mins/day = 20	N/A	**Spatiotemporal** • Step length • Cadence • Gait speed	**Spatiotemporal** (n = 11) (pre-post) Mean step length (SD) (cm) Pre: 39.9 (10.1) Post: 43.5 (9.8) P = <0.008** Cadence (SD) (step/min) Pre: 115.6 (30.3) Post: 122.1 (31.2) P = 0.127 Gait speed (SD) (m/min) Pre: 47.1 (18.4) Post: 54.7 (22.2) P = 0.019*	Single HAL use is likely to be safe and may be capable of immediate improvement in gait and PROM in children with CP
**Orekhov et al. (2020) USA [33]**	Case Series	6	Age Range: 9–31 yrs F: 1 M: 5 Diagnosis: Ambulatory individuals with CP GMFCS: Level I: 3 Level II: 1 Level III: 2 Co-Intervention: No	Exoskeleton: Battery-powered, lightweight ankle exoskeleton n Rx = 3	N/A	**Spatiotemporal** • Gait speed **Metabolic cost of transport**	**Spatiotemporal** Gait speed • Low assistance compared to baseline: Increased 5.9% ± 2.5% (p = 0.034*) • Training-tuned compared to baseline: Increased 3.9% ± 1.9% (p = 0.050*) • High assistance compared to baseline: Increased 6.9% ± 2.4% (p = 0.018*) • Walking with the exoskeleton unpowered did not significantly affect speed compared to baseline **Metabolic cost of transport** • Low assistance compared to baseline: No change (p = 0.232) • Low assistance compared to unpowered condition: No change (p = 0.066) • Training-tuned compared to baseline: No change (p = 0.130) • Training-tuned compared to unpowered condition: Decreased by 15.3% (p = 0.010**) • High assistance compared to baseline: Decreased 8.5% (p = 0.042*) • High assistance compared oi unpowered condition: Decreased 17.6% (p = 0.001**)	A powered exoskeleton may improve gait via potential increase in walking speed and potential reduction in both metabolic cost of transport and soleus muscle activity in individuals with CP
**Smania et al. (2011) Italy [34]**	Case Study	1	Age: 11 yrs F: 0 M: 1 Diagnosis: Spastic tetraplegic CP GMFCS: Level IV: 1 Co-Intervention: No	Exoskeleton: NF-Walker n Rx = 1	N/A	**Spatiotemporal** • Gait speed • **Metabolic CostEnergy Cost**	**Spatiotemporal** Gait speed Baseline: N/A • Experimental condition: 0.12 (m.s-1) **Metabolic Cost** Oxygen uptake (V’O2 L.min-1) • Baseline: 7.1 • Experimental condition: 15.5 Carbon dioxide output (V’CO2 L-min-1) • Baseline: 215.5 • Experimental condition: 468.3 Pulmonary ventilation (V’E L.min-1) • Baseline: 13 • Experimental condition: 26.4 Heart rate (HR) • Baseline: 134.3bpm • Experimental condition: 162.4bpm **Energy Cost** Baseline: N/A Experimental condition: 24.7 (j.kg-1.m-1) No reporting of P Values	Wearing NF-Walker exoskeleton may make gait possible in non-ambulatory children with CP
**Takahashi et al. (2018) Japan [44]**	Case Series	14	Mean Age: 15.6 yrs F: 4 M: 10 Diagnosis: Spastic (13), Athetosis (1) CP types GMFCS: Level I: 3 Level II: 3 Level III: 8 Co-Intervention: No	Exoskeleton: The Hybrid Assistive Limb (HAL) CYBERDYNE n Rx = 1 mins/day = 20	N/A	**Spatiotemporal** • Step length • Cadence • Gait speed	(*p<0.05) 95% confidence interval [CI] **Spatiotemporal** Gait speed (m/s) Pre: 0.71 ± 0.35 Post: 0.83 ± 0.45* (p = <0.05) [CI] 0.03 to 0.21 Mean step length (m) Pre: 0.44 ± 0.12 Post: 0.47 ± 0.13* (p = <0.05) [CI] 0.00 to 0.07 Cadence (step/second) Pre: 1.53 ± 0.50 Post: 1.66 ± 0.70 (p = >0.05) [CI] −0.07 to 0.34	Single HAL use is likely to be safe and may be capable of immediate improvement in gait in individuals with CP
**Ueno et al. (2019) Japan [43]**	Case Series	8	Mean Age: 18.2 F: 4 M: 4 Diagnosis: Bilateral spastic CP GMFCS: Level III: 3 Level IV: 5 Co-Intervention: No	Exoskeleton: Hybrid Assistive Limb–Treatment (HAL-T) n Rx = 8 4/52	N/A	**Spatiotemporal** • Step length • Cadence • CGS	**Spatiotemporal** (pre-post) Step length (m) Pre: 0.24 ± 0.10 Post: 0.34 ± 0.10 (p = 0.020*) (95% confidence interval [CI]: 0.02–0.16m) Cadence (steps/min) Pre: 74.8 ± 23.1 Post: 92.8 ± 25.8 (p = 0.015*) (95% confidence interval [CI]: 4.7–31.4 steps/min) CGS (m/s) Pre: 0.31 ± 0.17 Post: 0.51 ± 0.16 (p = 0.006**) (95% confidence interval [CI]: 0.07–0.31 m/s)	HAL-T use is likely to be safe and may improve gait in children and young adults with CP

**KEY:** GMFCS = Gross Motor Function Classification System, CGS = comfortable gait speed, 10MWT = ten metre walk test, 6MWT = six minute walk test, ROM = range of motion, PROM = Passive range of motion, LL = lower limb, EMG = electromyography, iEMG = integrated electromyography, SCALE = Selective Control Assessment of Lower Extremity, PCI = Physiological Cost Index, GDI = Gait Deviation Index, GPS = Gait Profile Score, GMFM-88 = Gross Motor Function Measure– 88 Items, IC = initial contact, CGS = comfortable gait speed, n = number of, Rx = treatment, CP = cerebral palsy, M = male, F = female, P1 = patient one, P2 = patient two, P3 = patient three, P4 = patient four, R = right, L = left, MWS = maximum walking speed, SWS = self-selected walking speed, N/A = not applicable

* = results are statistically significant (p = ≤0.05)

** = results are statistically significant (p = ≤0.01).

### Participant characteristics

The overall number of participants from all the included studies was 82 and varied between 1–19 across individual studies. Age of the participants ranged from 5–31 years and there was a gender bias towards males. Frequently reported participant characteristics included the clinical diagnosis of CP with classifications ranging from I-IV on the GMFCS scale.

### Types of intervention

Seven of the 13 studies with a total of 61 participants [39, 41, 43, 44, 46–48] used the Hybrid Assistive Limb (HAL) and two studies with a total of 6 participants [31, 42] used the CPWalker robotic platform. The remaining studies totaling 17 participants used four different robotic devices including a lower-extremity exoskeleton [32], the Wearable Ankle Knee Exoskeleton (WAKE-up) [45], a battery-powered, lightweight ankle exoskeleton [33] and the NF-Walker exoskeleton [34]. The number of total interventions ranged from a solitary intervention to 16 sessions. Five studies [34, 41, 44–46] utilised a solitary session while three studies [31, 42, 43] utilised two sessions per week. Two studies [33, 39] had three sessions per week and Matsuda et al. [46] had 2–4 sessions per week with a total of 12 interventions. The studies conducted by Lerner, Damiano and Bulea [32] and Mataki et al. [48] had an average of one session per fortnight and per month, respectively. The shortest session duration was 10–20 minutes by Matsuda et al. [46] while Lerner, Damiano and Bulea [32] had the longest session duration of 2–3 hours. Four studies [41, 43, 44, 47] had session durations of 20 minutes. Three studies [39, 42, 48] had session durations of 60 minutes. Mileti et al. [45] and Bayon et al. [31] had session durations of 30 minutes and approximately 75 minutes, respectively. Two studies [33, 34] did not report on session duration at all. Nakagawa et al. [39] included 10 minutes of single-leg extension-flexion motion and standing and seated exercises as pre-training into their study design. Study duration ranged from 1 day [34, 41, 44–46] to 8–12 weeks [32]. Three studies [39, 43, 47] had a duration of four weeks and an additional three studies had durations of five weeks [42], eight weeks [31] and 4–8 weeks [48]. Orekhov et al. [33] did not report on the duration of study. Table 3 provides an overview of the intervention parameters.

**Table 3:** Intervention parameters.

Study	Type of Robotic Device	Overall frequency/ per week	Duration	
			Time	Weeks
Bayon et al. (2016) [42]	The CPWalker robotic platform	10 sessions in total • 2 sessions per week	60min sessions consisted of: • Walking in straight lines on an even surface at a hospital facility using CPWalker • 10min for setup • Rest time: Not reported	5 weeks
Bayon et al. (2018) [31]	The CPWalker robotic platform	16 non-consecutive sessions in total	Sessions consisted of: • 10–15min warm-up • 60min gait training using CPWalker • 3min independent gait as cool-down • Rest time: Not reported	8 weeks
Lerner, Damiano, Bulea (2017) [32]	Lower-extremity exoskeleton	6 sessions in total	2–3 hour sessions consisted of: • Gait training using exoskeleton • Rest time: Not reported	8–12 weeks
Mataki et al. (2018) [48]	The Hybrid Assistive Limb (HAL)	2 sessions in total	60min sessions consisted of: • 30min of gait training using HAL • 20min for attachment and detachment of HAL • 10min of rest	4–8 weeks
Matsuda et al. (2018a) [46]	The Hybrid Assistive Limb (HAL)	1 session in total	10-20min session consisted of: • Gait training using HAL over a 50-200m distance • Distance was dependent on participant condition, including fatigue, facial expression and pulse • Rest time: Not reported	1 day
Matsuda et al. (2018b) [47]	The Hybrid Assistive Limb (HAL)	12 sessions in total • 2–4 sessions per week	20min sessions consisted of: • Gait training using HAL • Rest time: Excluded in session duration • Rest was dependent on participant condition, including fatigue, facial expression and pulse	4 weeks
Mileti et al. (2016) [45]	The Wearable Ankle Knee Exoskeleton (WAKE-up)	1 session in total	30min session consisted of: • 5min of gait training using WAKE-up before trial • Trial involved participants walking at a self-selected speed through a 10m pathway five times with and without the exoskeleton. • Rest time: Seated breaks between repetitions were permitted to resolve fatigue	1 day
Nakagawa et al. (2019a) [39]	The Hybrid Assistive Limb (HAL)	12 sessions in total • 3 sessions per week	60min sessions consisted of: • 30min of gait training using HAL • 20min for attachment and detachment of HAL • 10min of pre-training, including single-leg extension-flexion motion and standing and sitting exercise • Total gait training: 238min • Mean gait training: 19.9min per session • Rest time: Not reported	4 weeks
Nakagawa et al. (2019b) [41]	The Hybrid Assistive Limb (HAL)	1 session in total	20min session consisted of: • Gait training using HAL • Rest time: Included in session duration	1 day
Orekhov et al. (2020) [33]	A battery-powered, lightweight ankle exoskeleton	3 sessions in total	1^st^ Session consisted of: • Participants performing baseline, unpowered, and exoskeleton-assisted walking assessments • Gait training using exoskeleton 2^nd^ Session consisted of: • Gait training using exoskeleton 3^rd^ Session consisted of: • Participants performing baseline, unpowered, and exoskeleton-assisted walking assessments • Gait training using exoskeleton ➢ Mean total exoskeleton acclimation time: 96.7min ➢ Rest time: Not reported	Not reported
Smania et al. (2011) [34]	The NF-Walker exoskeleton	1 session in total	Session consisted of: • Participants performing the 2MWT and the 10MWT wearing the exoskeleton in the non-actuated condition and in the actuated condition • Rest time: Not reported	1 day
Takahashi et al. (2018) [44]	The Hybrid Assistive Limb (HAL)	1 session in total	20min session consisted of: • Gait training using HAL • Rest time: Not reported	1 day
Ueno et al. (2019) [43]	The Hybrid Assistive Limb (HAL)	8 sessions in total • 2 sessions per week	20min sessions consisted of: • Gait training using HAL walking on a 26m looped track • Rest time: Excluded in session duration • Rest was dependent on complaints of fatigue or manifested gait disturbances	4 weeks

**KEY:** 10MWT = ten metre walk test, 2MWT = two minute walk test, min = minutes.

### Outcomes

A summary of outcomes from across the studies are highlighted in Table 4. Of the 13 included studies, spatiotemporal parameters and energy expenditure were measured using eight outcome measures. This highlights the diverse outcome measures used to evaluate the effect of PoLLE on primary (spatiotemporal) and secondary (energy expenditure) outcomes.

**Table 4:** Summary of outcomes.

Study	Primary Outcomes				Secondary Outcomes			
	Spatiotemporal Parameters				Energy Expenditure			
	Step length	Stride length	Cadence	Mean velocity	PCI	Energy Cost	BORG	Metabolic Cost
Bayon et al. (2016) [42]	↑(+)?	NR	↑(+)?	↑(+)?	NR	NR	NR	NR
Bayon et al. (2018) [31]	↑(+)?	NR	↑(+)?	↑(+)?	↓(+)?	NR	NR	NR
Lerner, Damiano, Bulea (2017) [32]	ND	NR	ND	ND	NR	NR	NR	NR
Mataki et al. (2018) [48]	NR	↑(+)?	↑(+)?	↑(+)?	NR	NR	NR	NR
Matsuda et al. (2018a) [46]	NR	Pre-post: ND During: ↓(-)*	Pre-post: ND During: ↓(-)*	Pre-post: ND During: ↓(-)*	NR	NR	NR	NR
Matsuda et al. (2018b) [47]	SWS: ↑(+)? MWS: ↑(+)?	NR	SWS: ↑(+)* MWS: ↑(+)*	SWS: ↑(+)* MWS: ↑(+)?	(↓(+)?	NR	NR	NR
Mileti et al. (2016) [45]	↑(+)*	↑(+)*	↓(-)*	ND	NR	NR	NR	NR
Nakagawa et al. (2019a) [39]	↑(+)?	NR	NR	↑(+)?	NR	NR	NR	NR
Nakagawa et al. (2019b) [41]	↑(+)*	NR	↑(+)?	↑(+)*	NR	NR	NR	NR
Orekhov et al. (2020) [33]	NR	NR	NR	Low assistance compared to baseline: ↑(+)* Training-tuned compared to baseline: ↑(+)* High assistance compared to baseline: ↑(+)*	NR	NR	NR	Low assistance compared to baseline: ND Low assistance compared to unpowered condition: ND Training-tuned compared to baseline: ND Training-tuned compared to unpowered condition: ↓(+)* High assistance compared to baseline: ↓(+)* High assistance compared to unpowered condition: ↓(+)*
Smania et al. (2011) [34]	NR	NR	NR	↑(+)?	NR	↑(-)?	NR	↑(-)?
Takahashi et al. (2018) [44]	↑(+)*	NR	↑(+)?	↑(+)*	NR	NR	NR	NR
Ueno et al. (2019) [43]	↑(+)*	NR	↑(+)*	↑(+)*	NR	NR	NR	NR

**KEY:** NR = no results (either not reported by the researchers or the authors did not provide adequate information to draw conclusions, i.e. no baseline measures), ND = no difference, ↓ = reduction with intervention, ↑ = increase with intervention

* = results are statistically significant (p = ≤0.05)

** = results are statistically significant (p = ≤0.01),? = significance not reported, + = results are positive for intervention,— = results are negative for intervention, PCI = Physiological Cost Index, IC = initial contact, CGS = comfortable gait speed, n = number of, Rx = treatment, CP = cerebral palsy, P1 = patient one, P2 = patient two, P3 = patient three, P4 = patient four, R = right, L = left, MWS = maximum walking speed, SWS = self-selected walking speed.

### Spatiotemporal parameters

All 13 studies investigated the effect of PoLLE on gait speed or velocity. Ten studies reported an increase in mean velocity as a result of the intervention. Of these studies, findings from five studies [33, 41, 43, 44, 47] were statistically significant while five studies [31, 34, 39, 42, 48] did not report data on statistical significance. There was no difference in mean velocity in the remaining three studies [32, 45, 46]. Ten studies measured change in cadence from robotic device use. Seven studies found cadence increased because of the intervention, with both Matsuda et al. [47] and Ueno et al. [43] reporting statistically significant findings. Nakagawa et al. [41] reported a non-statistically significant (P = 0.127) increase in cadence as did Takahashi et al. [44]. While three studies [31, 42, 48] reported an increase in cadence, this was not supported with data on statistical significance. Mileti et al. [45] found a statistically significant decrease in cadence in all three participants following gait training using the WAKE-up exoskeleton. The remaining two studies [32, 46] found no difference in cadence. With regards to step length, nine studies investigated this [31, 32, 39, 41–45, 47]. With the exception of Lerner, Damiano and Bulea [32] who did not report any change, all the other eight studies found improvements in step length. Four studies [41, 43–45] had a statistically significant increase in step length, while three studies [31, 39, 42] did not support their findings with statistical significance. Matsuda et al. [47] found a non-statistically significant increase in step length for both self-selected walking speed (P = 0.078) and maximum walking speed (P = 0.739) in participants using the HAL exoskeleton. Three studies measured change in stride length from robotic device use. Mileti et al. [45] had a statistically significant increase in stride length in two out of three participants from WAKE-up exoskeleton use. Mataki et al. [48] also found an increase in stride length after use of their robotic device however no p-values were reported. The remaining study conducted by Matsuda et al. [46] found no difference between pre and post data in stride length.

Two studies conducted by the same concentration of authors [46, 47] utilising HAL reported contradictory findings from comparable populations. Matsuda et al. [46] found no difference in pre-post results in gait speed, stride length and cadence. However, there was a statistically significant reduction in all three gait parameters during exoskeleton use. Conversely, Matsuda et al. [47] had an increase in pre-post results for gait speed, step length and cadence for self-selected and maximum walking speeds. The improvement was statistically significant in cadence for both walking speeds and gait speed for self-selected walking speed. The difference in results may be explained by the amount of gait training using the robotic device. The participants in Matsuda et al.’s [46] study had a solitary intervention of 10–20 minutes whilst subjects in Matsuda et al.’s [47] research undertook 12 sessions of 20 minutes which is likely to have led to familiarisation of the robotic device and a positive training effect. The findings of Takahashi et al. [44] and Nakagawa et al. [41] again from this same concentration of authors, show improvement in gait parameters is possible after a solitary intervention of 20 minutes. Takahashi et al. [44] had comparable participants to Matsuda et al. [46] and Matsuda et al. [47] with a statistically significant increase in pre-post gait speed and step length, as well as a non-statistically significant increase in cadence following gait training with HAL. Nakagawa et al. [41] also found improvement in these gait parameters with a statistically significant increase in step length and gait speed, however the subjects were younger in their study, approximately half the age of the other participants [44, 46, 47]. Nakagawa et al. [41] utilised a newly developed version of HAL which is less than half the weight of the other model and more suitable for shorter and lighter children.

### Energy expenditure

Five studies measured the effect of powered exoskeletons on energy expenditure. Two used the PCI [31, 47], two measured the metabolic cost of transport (respiratory and heart parameters) [33, 34] and one used the Borg Scale [39]. Orekhov et al. [33] found statistically significant improvements in the metabolic cost of transport in training tuned assistance (p = 0.010) and high assistance (p = 0.001) compared to unpowered exoskeletons. Statistically significant improvements were also found in high assistance (p = 0.042) compared to baseline conditions. No statistically significant differences were found with low assistance compared to baseline or unpowered assistance as well as training-tuned compared to baseline. Smania et al. [34] also measured the metabolic cost of transport including respiratory (O2 and CO2) measures with the addition of heart rate measures. The results from Smania et al. [34] demonstrated a negative impact on both respiratory and heart parameters from baseline to experimental conditions that was reflected when compared to reference normative data. No p-values were provided in this study. PCI was measured in two studies with Bayon et al [31] reporting all participants showed increased levels of oxygen cost measured by heart beats per minute during exercise compared to after. Matsuda et al. [47] measured before and after with the collective results from the five participants having a non-statistically significant (P = 0.405) decrease in levels of oxygen cost after the intervention compared to before the intervention. Nakagawa et al. [39] used the Borg Scale to measure the degree of fatigue recording a mean value of 11.9 that ranged between 11–13 (between light and somewhat hard).

### Safety

Of the 13 studies included, seven measured issues related to safety with the use of robotic devices. Five studies [34, 39, 44, 47, 48] reported no adverse effects while two studies by Nakagawa et al. [41] and Ueno et al. [43] reported minor adverse effects. Nakagawa et al. [41] reported a lower limb skin rash in two participants, lower limb sores in one participant and fear/crying in one participant. A study by Ueno et al. [43] also reported minor adverse effects that included skin peeling in three participants and a toenail groove breaking in one participant. These minor adverse effects were resolved across all the included studies with no long-term or serious adverse effects reported.

### Summary of results

Collectively, there is some consistent positive evidence to indicate that PoLLE may be effective in improving spatiotemporal parameters in gait. Ten out of the 13 studies found an increase in mean velocity as a result of the intervention [31, 33, 34, 39, 41–44, 47, 48], although only five of these findings were supported with statistical significance [33, 41, 43, 44, 47].

Seven of the ten studies which measured cadence reported an increase [31, 41–44, 47, 48], and this was supported by two studies with statistically significant findings [43, 47]. Eight studies reported improvements in step length [31, 39, 41–45, 47], albeit only some supported with statistical significance [41, 43–45], whereas Lerner, Damiano and Bulea [32] found no difference. Stride length was measured in three studies [45, 46, 48], two of which the parameter increased [45, 48], with Mileti et al. [45] reporting statistical significance. Five studies measured the effects of powered exoskeletons on energy expenditure [31, 33, 34, 47] with mixed findings. Three studies [31, 33, 47] supported a reduction in energy expenditure, although findings by Matsuda et al. [47] and Bayon et al [31] are not supported by statistical significance. Smania et al.’s [34] findings contradict these findings, as the use of exoskeletons required higher energy expenditure during the use of the device in comparison to the healthy aged matched participants. With regards to safety, only a handful of studies measured any adverse effects, which identified only minor and short-term concerns.

### NHMRC FORM framework

Table 5 provides an overview of synthesis of the results using the NHMRC FORM framework There are some consistent positive findings, from an emerging evidence base, for spatiotemporal parameters, and safety of PoLLE. While this is encouraging, caution is required due to the low level of, and heterogeneity within, the evidence base which was as a result of variability in the intervention designs and parameters (type of robotic device used; number, frequency, and duration of interventions), outcome measures used and varied study protocols (such as follow up periods).

**Table 5:** NHMRC FORM framework analysis.

Component	Grade	Comments
1. Evidence Base	D–Poor *Level IV studies*, *or level I to III studies with high risk of bias*	13 studies All case studies or case series–Level IV studies 84 individuals with CP across all studies
2. Consistency	C–Satisfactory *Some inconsistency reflecting genuine uncertainty around clinical question*	Consistent study designs– 10 case studies and three case series Age range 5-31yrs Variable dosage of intervention Variable exoskeleton designs Varied outcome measures Inconsistent reporting of statistical significance Consistent findings of positive outcomes for spatiotemporal parameters and safety Inconsistent findings for energy expenditure
3. Clinical Impact	D–Poor *Slight or poor*	Five studies reported on safety Two of the studies reported minor adverse effects Intervention protocols generally described adequately, however intervention parameters not justified Most of the studies showed improvements in spatiotemporal parameters, however mixed findings for energy expenditure Lack of reporting of clinical meaningful difference of outcomes No comparators to other treatments Much of the evidence comes from three groups of researchers
4. Generalisability	C–Satisfactory *Population/s studied in the body of evidence differ to the target population guideline but it is clinically sensible to apply this evidence to the target population*	The population across the included studies covers a proportion of the target population of this systematic review and could not cover the entire scope. Age range 5–31 years Various types of CP however includes a higher proportion of spastic diplegia GMFCS classifications ranging from I-IV, however includes a higher proportion of Levels III-IV Inclusion of both genders, with a slightly higher proportion of males Most studies did not describe their sample in detail, including their specific impairments The evidence base lacks clarity in terms of reporting co-interventions and its impact on outcomes All studies performed in a clinical setting Seven studies conducted in Japan, two in Spain, two in Italy and two in the USA
5. Grade of recommendations	C–Satisfactory *Body of evidence provides some support for recommendation(s) but care should be taken in its application*	While there were some consistent positive findings amongst spatiotemporal outcomes and safety, the evidence base is heterogenous in its intervention parameters, exoskeleton designs and outcome measures

## Discussion

Robotic therapies are newly emerging technologies that have become popular in the management of neurological conditions. To date, there has been no systematic review which has evaluated the evidence of PoLLE use in individuals with CP. Therefore, the purpose of this systematic review was to identify the best available evidence on the effectiveness for PoLLE use on gait in individuals with CP. This systematic review identified 13 eligible studies (ten case studies and three case series). Collectively, the body of evidence indicates that there is some consistent positive evidence on the effectiveness of PoLLE in improving gait in individuals with CP with minimal adverse effects. While this is a positive and encouraging finding for an emerging technology, it should be acknowledged that the included studies were of low level and low methodological quality. Methodological concerns related to sampling bias, variable reporting of the statistical significance of results and heterogeneity in intervention designs and parameters, and outcome measures. Therefore, caution is required when interpreting these results.

### Spatiotemporal parameters

The use of PoLLE for improving gait in individuals with CP produced positive results across several spatiotemporal parameters, highlighting the potential role for PoLLE in the management of these patients. These findings have been supported by previous research which have explored the effectiveness of robotic therapies on gait in various groups of neurological conditions. For example, a systematic review on the use of robotic gait training using the Lokomat or Gait Trainer GT I in individuals with CP found consistent statistically positive results on gait speed and step length [1]. These findings are not, however, universally shared as another systematic review [11] identified inconsistent results for gait speeds.

A scoping review on the effectiveness of PoLLE use in post-stroke patients also reported mixed results [24]. While two pre-post studies in sub-acute stroke patients demonstrated improvements in gait speed following a handful of therapy sessions, a randomised controlled trial with the same patient population did not report any significant difference between the PoLLE use and conventional therapy for gait speed [24]. The effects of PoLLE were far less apparent in patients with chronic stroke. Louie and Eng [24] suggest that this may be likely due to chronic stroke patients being ambulatory and may benefit from more unconstrained gait practice with greater variability. These findings complement the results from this systematic review as participants with a higher GMFCS level (Level III or IV) demonstrated the greatest improvements in outcomes (as identified in [31, 33]). A possible explanation for this is that individuals with a greater level of neuromuscular impairment have lesser baseline spatiotemporal measurements, which may provide greater capacity for improvement during walking with powered assistance [33]. This may suggest that individuals with the greatest potential to demonstrate benefits from PoLLE gait training are individuals with CP that have some existing capacity to ambulate.

### Energy expenditure

While PoLLE had a positive impact on spatiotemporal parameters, the results on the effects of energy expenditure were mixed. There are two likely explanations for this finding. First, the research by Smania and colleagues [34] was a GMFCS Level IV case study, and therefore energy expenditure comparisons are drawn from the participant’s baseline non-ambulatory state. Second, the participant’s energy expenditure during the PoLLE training was significantly higher than age-matched healthy children, which may be due to the participant not being accustomed to autonomous locomotion [38]. While previous research has reported that wearing assistive robotic technology reduces energy expenditure, this has been limited to a single population group. For example, one study demonstrated that the use of the ReWalk exoskeleton decreased energy expenditure across a range of parameters when compared to the use of a knee ankle foot orthosis in patients with spinal cord injury (SCI) [49]. Similarly, a review of the literature reported that the use of powered and hybrid orthoses does reduce energy expenditure in comparison with conventional orthosis in patients with SCI [50].

The mixed findings of our systematic review on energy expenditure outcomes may also be attributed to the variation in exoskeleton designs and participant characteristics. The ratio of the exoskeleton mass to the participant’s mass must be small to provide energy efficiency benefits [33]. A systematic review on the effect on energy consumption of robotic therapies, including end-effectors, treadmill-based exoskeletons and wearable exoskeletons in stoke and SCI patients, found that energy outcomes depended on robot type, walking speed, amount of body weight support or amount of effort [51]. Lefeber and colleagues [51] demonstrated that participants had a significantly lower energy expenditure when walking with a powered exoskeleton compared with treadmill walking-only in conditions of higher gait speed and larger step lengths [51]. This may suggest that walking with a more normal gait pattern correlates with increased energy efficiency in these individuals. These findings align with that of Lerner and colleagues [32] who reported that improved knee extension in walking with the PoLLE may ease the energy burden of walking as a flexed posture increases the energy consumption of gait [32].

### Safety

As PoLLEs are an emerging intervention which use rapidly developing technologies, to date much of the focus has been on demonstrating its effectiveness. Only a handful of studies reported any data regarding safety (seven of the 13 included studies [34, 39, 41, 43, 44, 47, 48]), of which only two studies reported minor adverse effects such as skin rash and lower limb sores, which resolved quickly [41, 43]. Skin abrasions may occur with exoskeleton use due to the correction of gait patterns during repetitive walking [43]. While these minor adverse effects are encouraging, previous research has highlighted opportunities for more serious adverse effects such as falls, fractures and soft tissue injuries [23, 52]. Therefore, ongoing research with long term follow up is required to systematically gather and evaluate the safety of PoLLEs.

### Limitations

As with any research, this systematic review has limitations. Even though this systematic review identified 13 studies, many of these were pilot studies. This is not unusual though, as pilot studies are ideal designs to test emerging interventions such as PoLLEs. Accordingly, all but one study included in this systematic review were published in the last four years. Nevertheless, methodological concerns need to be acknowledged. Due to the nature of the study designs, studies had small sample sizes, ranging from a single person case study [34, 39, 48] to 19 participants [41]. There was heterogeneity in terms of participant classification using GMFCS levels. There was a greater proportion of participants included who were rated Level II (n = 19) and III (n = 33) on the GMFCS. Only 11 Level I participants were included, 18 Level IV participants and there were no Level V participants. While there was no clear justification as to why there was a focus on participants who were classified as Level II and III on the GMFCS, it might indicate that PoLLEs may be best suited to these patient populations. Another limitation of the study design was the lack of a true control group and hence concerns regarding maturation bias. Due to the complexity of measuring gait, there was variability in how this was measured with a plethora of outcome measures used and with minimal reporting of psychometric properties.

Co-interventions were not avoided, with many participants continuing to receive their conventional therapies alongside the PoLLE intervention. Bayon et al. [31] argued that standard physiotherapy interventions would not affect the results of their study as the participants had been receiving conventional physiotherapy for years previously and had made no significant functional improvement. Furthermore, from a clinical practice perspective, PoLLEs could be considered as an adjunct to conventional therapies. Therefore, while co-intervention bias needs to be acknowledged, the use of PoLLEs as a co-intervention may reflect what occurs in everyday clinical practice contexts.

The role of publication and language bias within this systematic review should be acknowledged even though it was underpinned by best practices in the conduct and reporting of systematic reviews (PRISMA). A search of grey literature, secondary sources and pearling of reference lists was completed to avoid publication bias. Despite these efforts, some publications may have been missed due to the accessibility constraints, limited access to full research reports and the imprecise and complex nature of searching grey literature. Language bias must be considered as this systematic review search only included English-language publications. However, this was mitigated by an extensive and wide-ranging search strategy, which resulted in literature sourced from a range of countries where English is not the first language (Spain, Italy and Japan).

## Conclusion

### Implications for practice

With technology playing an increasing role in health care, there is potential for PoLLEs to play a role in the rehabilitation of people impacted by CP, SCI and other neurological conditions. This review has identified an emerging body of evidence which suggests that PoLLEs could improve gait in individuals with CP with minimal adverse effects. As PoLLEs are a new technology, the evidence base is in its infancy and continues to evolve. Therefore, these positive findings should be balanced with a range of factors which will influence implementation in clinical practice such as cost, resources and access to and availability of trained therapists. Firstly, given this is an emerging technology, PoLLEs may not be widely available and may be limited to some regions (such as metropolitan settings) and contexts (such as health services which are linked with research institutions as some PoLLEs continue to be tested). Secondly, as with any new intervention, health care professionals require adequate training and certification prior to use with patients and this process will require adequate time and resourcing. Finally, patient perspectives and preferences need to be carefully considered as PoLLEs may not be suitable for all individuals with CP as some may not be comfortable with the exoskeleton (due to its weight) and its attachments (such as power supply cables).

### Recommendations for future research

While the emerging evidence base has identified some support for the positive impact of PoLLEs on individuals with CP in terms of gait, given the low level and low-quality evidence, there are some methodological concerns. Future research should contribute to the evidence base across a range of areas. Further studies should develop a standardised set of parameters for intervention which can then be applied universally when exploring the effect of robotic therapy. This will ensure homogeneity between studies and allow for more accurate recommendations to be put forward. It is in this context single-case experimental designs might be useful when finetuning the optimal PoLLE designs and intervention parameters. Similarly, a core set of outcome measures could be developed and tested for psychometric properties in terms of validity and reliability. This would then form the base set of measures used by all research when demonstrating the effectiveness of robotic therapy. These measures particularly need to be assessed for a longer-term post-intervention to discover the long-term effects of PoLLEs. These measures could be extended to include safety, especially over long-term use. Furthermore, future research could compare PoLLEs to other interventions for CP. This will help to provide a cost-benefit analysis for the use of PoLLEs to ensure the health and physical benefits of exoskeleton use are commensurate to the resources utilised. Finally, as the evidence base evolves, more robust and mature research designs such as randomised controlled trials can be used to further demonstrate the impact of PoLLEs, including testing the effectiveness of different exoskeleton designs (e.g. single joint vs multiple joints).

## Supporting information

S1 Appendix PRISMA checklist. (DOCX)

S2 Appendix Medline search strategy. (DOCX)

S3 Appendix Medline search syntax. (PNG)
