# Supplementary material for: Effectiveness of powered exoskeleton use on gait in individuals with cerebral palsy: A systematic review
Source: PLoS One. 2021 May 26;16(5):e0252193. doi: 10.1371/journal.pone.0252193 (PMC8153467; doi:10.1371/journal.pone.0252193)
Supplement: S2 Appendix — (DOCX) [file pone.0252193.s002.docx]

**Medline Search Strategy**

| **1** | exp Cerebral Palsy/ |
| --- | --- |
| **2** | (cerebral pals* or cp or athetoid cerebral pals* or atonic cerebral pals* or dyskinetic cerebral pals* or dystonic-rigid cerebral pals* or dystonic rigid cerebral pals* or hypotonic cerebral pals* or spastic cerebral pals* or mixed cerebral pals* or ataxic cerebral pals* or monoplegic cerebral pals* or diplegic cerebral pals* or quadriplegic cerebral pals* or congenital cerebral pals* or rolandic type cerebral pals* or rolandic-type cerebral pals* or little* disease or spastic hemiplegi* or spastic diplegi* or spastic quadriplegi* or infantile cerebral pals* or monoplegic infantile cerebral pals* or diplegic infantile cerebral pals* or quadriplegic infantile cerebral pals* or brain pals* or brain paralys* or central pals* or central paralys* or cerebral paralys* or cerebral pares* or encephalopathia infantilis).mp. |
| **3** | exp Exoskeleton Device/ |
| **4** | (exoskelet* device* or exoskelet* robot* exoskelet* or power* exoskelet* or exoskelet*-type robot* or exoskelet* type robot* or exo-suit* or exosuit* or wearable robot* or robot*-suit or robot* suit* or exoskelet* assist* or exoskelet*-assist* or ortho exoskelet* or power* ortho* or power* gait ortho* or PGO or "hybrid assist* limb*" or "hybrid-assist* limb*" or HAL or power* armour* or power* armor* or power*-suit* or power* suit* or exo-frame* or exoframe* or hard-suit* or hardsuit* or rewalk or ekso* or cyberdyne or phoenix or indego or vanderbilt or rex* or CPwalker or CP-walker or "wear* ankle knee exoskelet*" or "wake-up exoskelet*" or "wake up exoskelet*" or elegs or mindwalker or "wearable power-assist* locomot" or "wearable power assist* locomot" or WPAL or exo-H2 or robin or "able exoskelet*" or "advanced reciprocating gait ortho*" or ARGO or "power* reciprocating gait ortho*" or "power* RGO" or arke or atlas or b-temia or keeogo or tibion or "honda walk* assist* device*" or "honda walk* assist*" or "X1 robot* exoskelet*" or mina or trexo*).mp. |
| **5** | Robotics/ |
| **6** | (robot* or robot* device* or robot* assist* or robot*-assist* or robot* based gait train* or robot*-based gait train* or robot* platform* or robot* ortho*).mp. |
| **7** | 1 or 2 |
| **8** | 3 or 4 or 5 or 6 |
| **9** | 7 and 8 |
