# Supplementary figures and images for: Effectiveness of powered exoskeleton use on gait in individuals with cerebral palsy: A systematic review
Source: PLoS One. 2021 May 26;16(5):e0252193. doi: 10.1371/journal.pone.0252193 (PMC8153467; doi:10.1371/journal.pone.0252193)

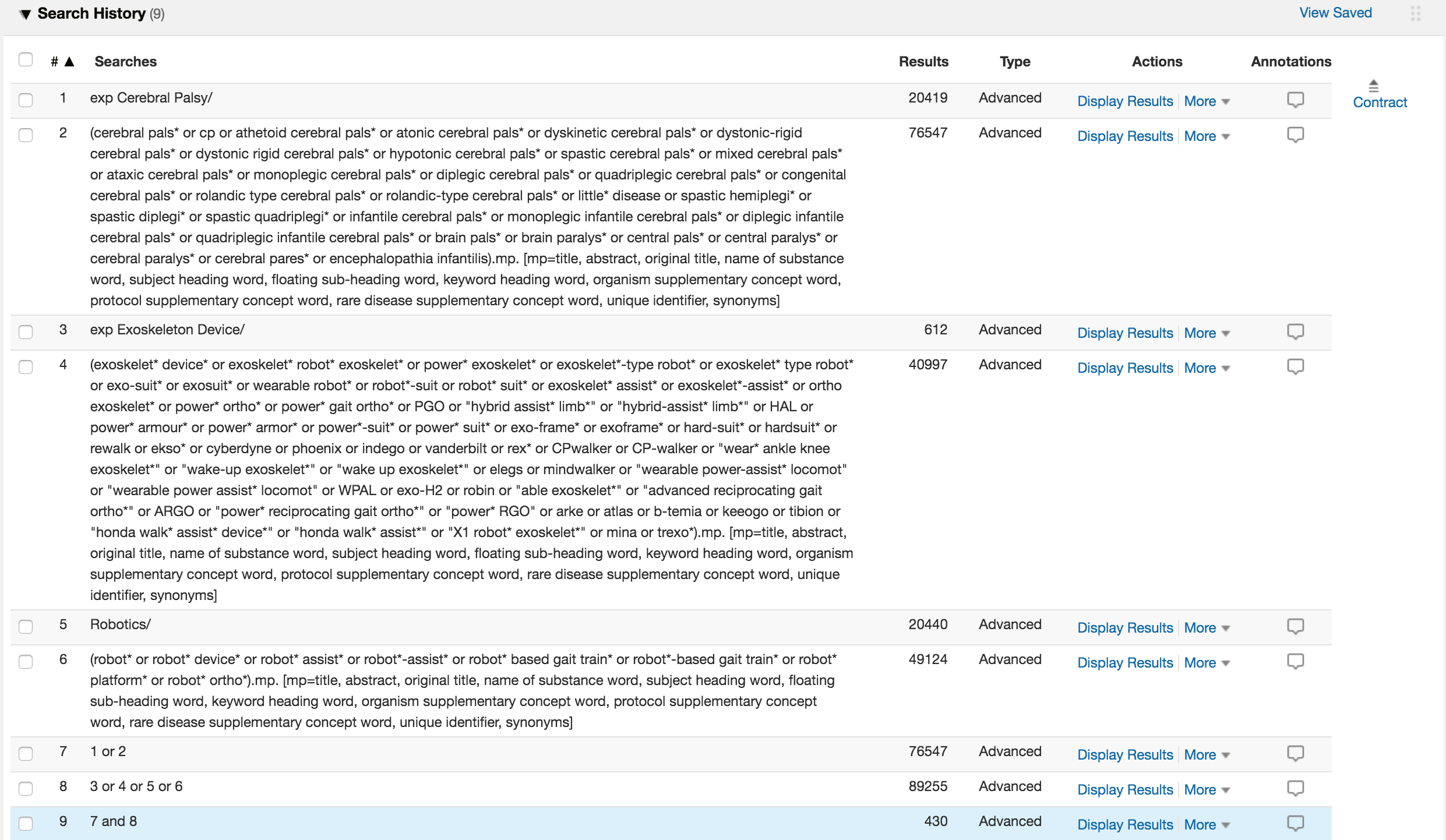

Supplement: S3 Appendix — (PNG) [file pone.0252193.s003.png]
